# Genome-Wide Analysis of Human Metapneumovirus Evolution

**DOI:** 10.1371/journal.pone.0152962

**Published:** 2016-04-05

**Authors:** Jin Il Kim, Sehee Park, Ilseob Lee, Kwang Sook Park, Eun Jung Kwak, Kwang Mee Moon, Chang Kyu Lee, Joon-Yong Bae, Man-Seong Park, Ki-Joon Song

**Affiliations:** 1 Department of Microbiology, the Institute for Viral Diseases and Korea Bank for Pathogenic Viruses, College of Medicine, Korea University, Seoul, Republic of Korea; 2 Department of Laboratory Medicine, College of Medicine, Korea University, Seoul, Republic of Korea; University of Malaya, MALAYSIA

## Abstract

Human metapneumovirus (HMPV) has been described as an important etiologic agent of upper and lower respiratory tract infections, especially in young children and the elderly. Most of school-aged children might be introduced to HMPVs, and exacerbation with other viral or bacterial super-infection is common. However, our understanding of the molecular evolution of HMPVs remains limited. To address the comprehensive evolutionary dynamics of HMPVs, we report a genome-wide analysis of the eight genes (N, P, M, F, M2, SH, G, and L) using 103 complete genome sequences. Phylogenetic reconstruction revealed that the eight genes from one HMPV strain grouped into the same genetic group among the five distinct lineages (A1, A2a, A2b, B1, and B2). A few exceptions of phylogenetic incongruence might suggest past recombination events, and we detected possible recombination breakpoints in the F, SH, and G coding regions. The five genetic lineages of HMPVs shared quite remote common ancestors ranging more than 220 to 470 years of age with the most recent origins for the A2b sublineage. Purifying selection was common, but most protein genes except the F and M2-2 coding regions also appeared to experience episodic diversifying selection. Taken together, these suggest that the five lineages of HMPVs maintain their individual evolutionary dynamics and that recombination and selection forces might work on shaping the genetic diversity of HMPVs.

## Introduction

Human metapneumovirus (HMPV) is a single-stranded RNA virus that belongs to the family *Paramyxoviridae* [1, 2]. Mostly in young children and the elderly, HMPV causes respiratory distress similar to that associated with human respiratory syncytial virus (hRSV), ranging from upper respiratory disease to bronchiolitis and pneumonia [3–5]. Based on the previous reports, HMPV infection appears to be seasonal [6], and co-infection with other respiratory pathogens is common [7–10]. However, there are still no vaccines or antivirals that can be used to treat HMPV infection [11, 12].

Among the eight genes (N, P, M, F, M2, SH, G, and L) of HMPVs, the genetic property of the membrane glycoproteins F and G defines two major lineages, A and B, which can be further classified into the four different lineages (A1, A2, B1, and B2) and two additional sublineages (A2a and A2b) [3, 13, 14]. Most of these lineage/sublineage strains have co-circulated, except for the A1 lineage that had been only detected until 2003 [15]. However, no specific lineages appear to have predominated among circulating HMPV strains [16, 17], and the dominant lineages vary year by year [18–21].

Previously, a close genetic relationship between HMPV and avian metapneumovirus (aMPV) subtype C was suggested by comparing sequence identity, genomic organization, and phylogenetic location [22–24]. A possible transmission hypothesis of the HMPV from avian species was then testified by analyzing the genomic sequences of N, F, and G coding regions and by determining serum antibody levels against the aMPV [25, 26]. Zoonotic cases of HMPV infection from humans to great apes were also reported [27–29]. Since then, various studies have assessed the genetic relationships between the HMPV lineages [18, 21, 30–32]. However, these all were based on the analyses of partial genes, and comprehensive understanding of evolutionary dynamics of HMPVs still remains limited.

As presented in the cases of RNA virus [10, 33–35], evolutionary forces, such as mutation and recombination, might have contributed to the genetic diversity of HMPVs. Through interactions or sequential actions, all of these forces might affect the accumulation of mutations arisen spontaneously or in response to the immune systems of infectees, which would eventually shape the evolutionary structure of HMPVs [36]. During this course of evolution, beneficial mutations are retained, but deleterious mutations are removed via purifying or negative selection [37]. For HMPV genes, negative selection force appeared to manage its effects on viral evolution, but only a limited number of residues has been detected as diversifying or positive selection sites, especially in the membrane glycoproteins G and F [18, 25]. Recombination is also considered an evolutionary force working on the genetic diversity of the *Pneumovirinae* viruses [38], and it was suspected in the aMPVs, based on the phylogenetic incongruence of the same viral genes [39]. However, there have been no confirmed cases of recombination reported in HMPVs.

A vaccine is the most effective prevention measure against infectious diseases [40]. Using computational approaches, antigenic clustering and transmission of some genetic signatures of other RNA viruses have been successfully demonstrated [41, 42]. Recently, a novel Baysian method of estimating phylogenetic signals against an uncertain prior condition has been suggested to investigate the genetic footprints of virulence and antigenic evolution of some viruses [43]. Given the ongoing diversifying progress of HMPVs, the computational methods may have crucial importance for developing effective medical arsenals against the circulation of divergent HMPV strains. When accompanied with the classical approaches of *in vitro* and *in vivo* virological testing, these may unveil scientific priorities much sooner for the development of vaccines and antivirals against HMPVs.

In this study, we analyzed 103 complete genome sequences of HMPVs using computational estimation methods of phylogenetic reconstruction, recombination, and natural selection. Based on the derived phylogenetic relationships and potential breakpoints of recombination, we suggested recombination as a possible mechanism underlying the evolutionary dynamics of HMPVs. We also provided the profiles of selection pressure analyses and discussed their evolutionary effects on the HMPV genetic diversity.

## Materials and Methods

### Sequence preparation

A total of 166 complete MPV sequences were downloaded from the Virus Pathogen Resource database (http://www.viprbrc.org/brc/home.spg?decorator=vipr). These were mainly contributed by two HMPV sequencing projects (BioProject PRJNA73051 and 237298). Two prototype HMPV sequences, NL/00/01 (GenBank accession number AF371337) and NL/99/01 (AY525842), were not included in this study because no collection dates were available from them. Among 166 complete genomes, the sequences from unknown hosts (n = 39), no collection date information (n = 2: DQ843658 and DQ843659), avian origins (n = 10: HG934338, KC915036, DQ009484, NC007652, EF199771, EF199772, AB548428, FJ977568, HG934339, and JF424833), and gorilla sequence (n = 1: HM197719) were removed at first. In addition, 11 sequences having missing nucleotide information in the protein coding regions (KF530158, missing 5749–6141 and 6640–7212 nucleotide regions; KF530160, 1091–1423 and 6244–7220; KF530165, 6670–7236; KF530166, 6332–7098; KF530177, 8074–8142; KF530181, 6716–7234; KF530182, 5862–7477 and 7911–8183; KF530183, 4428–4898 and 7446–7783; KF530185, 6814–6887; KF686741, 3212–3575 and 6355–7095; and KF686743, 6416–7152) were also removed. Hence, 103 complete sequences of HMPVs including a Korean HPMV KUMC-MP strain (KF516922) were prepared for genomic investigation. After initial alignment using the MUSCLE method in MEGA5.2 [44], datasets of the eight genes were established by extracting corresponding coding region sequences. Each dataset was then re-aligned using the MAFFT program (v7.130b) [45] and trimmed at the N- and C-terminal regions to ensure that each dataset retained the open reading frame or common protein coding regions. The stop codon in the C-terminal region and sequence irregularities found at the C-termini of the SH and G coding regions were also removed. The resulting HMPV datasets were assigned to nucleoprotein N (nucleotide region: 31–1,182; nucleotide length: 1,152), phosphoprotein P (1–885; 885), matrix protein M (1–762; 762), fusion glycoprotein F (1–1,617; 1,617), matrix protein-2 M2 (1–724; 724; for M2-1 coding region, 1–561; and for M2-2, 512–724), small hydrophobic protein SH (1–531; 531), attachment glycoprotein G (1–444; 444), and RNA-dependent RNA polymerase L (1–6,015; 6,015). The complete genome sequence set was constituted by concatenating from the N through the L genes together (N-P-M-F-M2-SH-G-L in order). The aligned genomic sequences are provided as S1 Data.

### Phylogenetic trees and evolutionary dynamics

Phylogenetic relationships, evolutionary rates (nucleotide, synonymous, and nonsynonymous substitutions/site/year), and the time (year) of the most recent common ancestor (tMRCA) were estimated using a time-framed Bayesian evolution analysis approach via a Markov Chain Monte Carlo (MCMC) inference method, implemented in the BEAST package (v1.8.1) [46]. The estimates were obtained based on the two tree models (Bayesian skygrid and Bayesian skyline) using the prior setting of the general time-reversal (GTR)+I+Γ substitution model and the lognormal relaxed molecular clock. The MCMC analysis ran for 200 million iterations, with sampling every 200 thousand iteration after 10% burn-in. Two independent runs for each dataset were combined and assessed to ensure their convergence in Tracer v1.6 [47]. The MCMC tree samples were used to infer the maximum clade credibility (MCC) trees for each dataset using TreeAnnotator v1.8.1. The MCC trees were then edited for better visualization using FigTree v1.4.2. Relative genetic diversity (N_e_τ: N_e_, the effective population size; τ, generation time) was also estimated by means of Bayesian skyline reconstruction to assess the population dynamics of each of the HMPV lineage sequences. The estimates were presented as mean values along with the lower and upper limits of the 95% highest probability density (HPD).

### Recombination and natural selection

Two models of recombination analysis, the single breakpoint of recombination (SBP) and genetic algorithms for recombination detection (GARD) methods, were used to screen potential breakpoints in the HMPV genomes in the condition of best-fit substitution models and beta-gamma site-to-site rate variation with four rate classes, using the web server of Datamonkey (http://www.datamonkey.org) together with the HyPhy package (v2.2) [48–50]. Based on the detected breakpoints, the nucleotide sequence sets were divided into pre- and post-breakpoint regions and used for the comparison of phylogenetic replacements using MEGA5.2 (maximum likelihood tree, GTR+I+Γ, 500 bootstrap replications) [44, 51]. Natural selection was also assessed using the same web server and HyPhy program. Site-specific pressures were analyzed by estimating the ratio of non-synonymous (dN) to synonymous substitutions (dS) at every codon using the single likelihood ancestor counting (SLAC) method employing the best-fit REV nucleotide substitution model (cutoff *p*-value, 0.05) [52]. The mixed effects model of evolution (MEME) method was also used to determine the sites of episodic and pervasive positive selection [53].

### Isolation of a HMPV from clinical nasopharyngeal specimens

Clinical nasopharyngeal specimens have been sampled from the patients at Korea University Medical Center (KUMC) Guro Hospital to screen the presence of various respiratory pathogens in the specimens, which would be exempted from the approval of Institutional Review Board of KUMC Guro Hospital. A HPMV strain, named as KUMC-MP, was isolated from one of the specimens and deposited to Korea Bank for Pathogenic Viruses, Korea University College of Medicine. From this strain, we obtained its complete genome sequence (GenBank accession number KF516922) by reverse transcription-PCR and used it in this study. The sequences of the primer sets were presented in S1 Table.

## Results

### Phylogenetic relationships of HMPV genomes and potential recombination events

**Fig 1:**
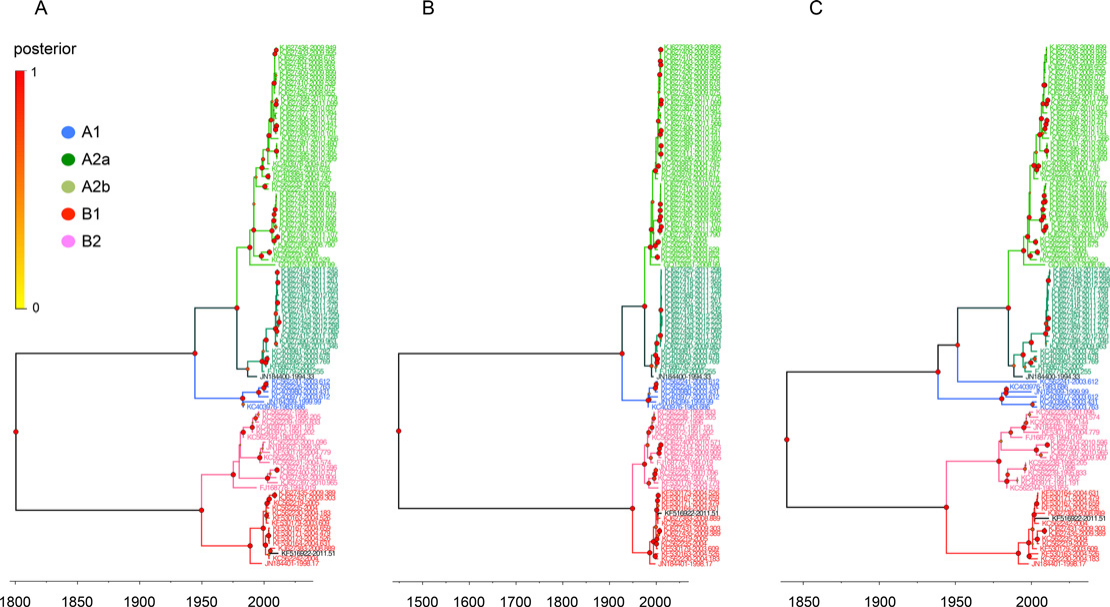
Phylogenetic relationships of HMPV N, P, and F gene sequences. The relative phylogenetic relationships of HMPV N (A), P (B), and F (C) gene sequences were defined in time-framed maximum clade credibility (MCC) trees. The five different colors represent each lineage (blue, A1; green, A2a; lime, A2b; red, B1; and pink, B2). The genes of JN184400 and KF516922 are colored deep green and black, respectively. The isolation time point of each sequence is expressed as the year fraction at the end of the sequence accession number. As the color of circles in the tree nodes, the size of circles in the node represents the posterior probability of their clustering (the bigger size demonstrates the higher probability).

**Fig 2:**
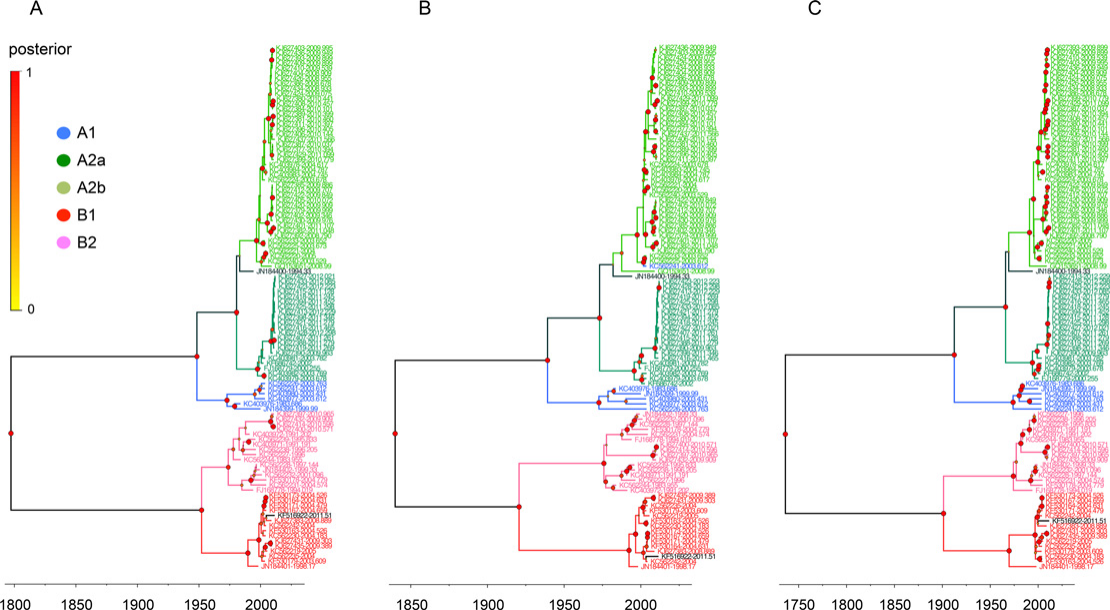
Phylogenetic relationships of HMPV SH, G, and L gene sequences. The relative phylogenetic relationships of HMPV SH (A), G (B), and L (C) gene sequences were defined in MCC trees. The five different colors represent each lineage (blue, A1; green, A2a; lime, A2b; red, B1; and pink, B2). The genes of JN184400 and KF516922 are colored deep green and black, respectively. Please see the Fig 1 legend for the information of isolation time points of sequences and posterior probability of tree branches.

**Fig 3:**
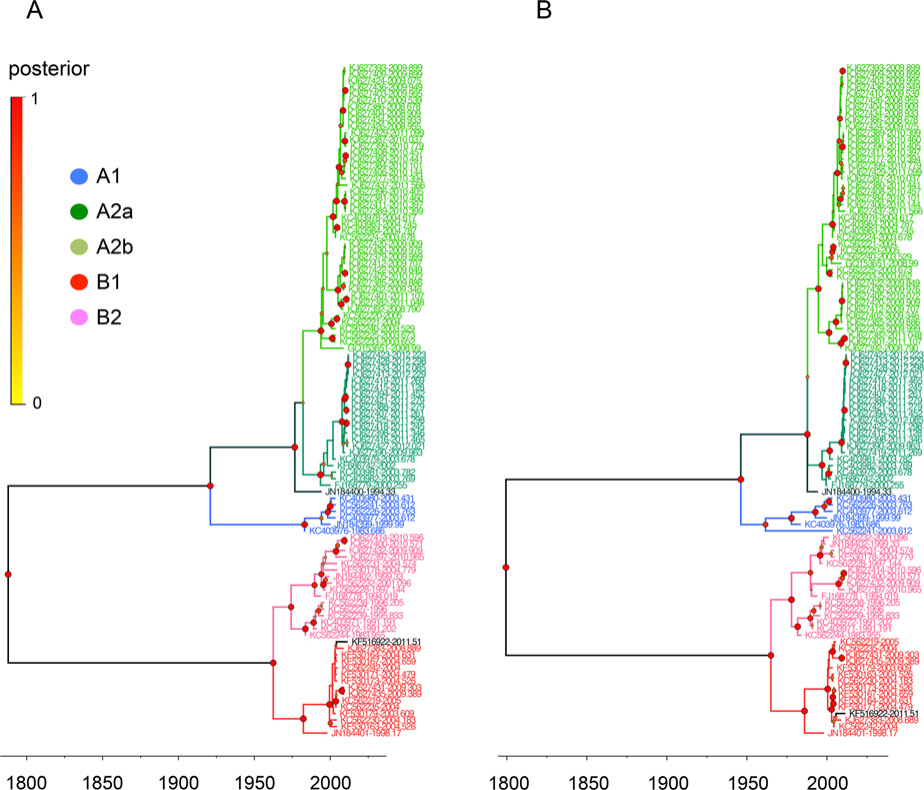
Phylogenetic relationships of HMPV M and M2 gene sequences. The relative phylogenetic relationships of HMPV M (A) and M2 (B) gene sequences were defined in MCC trees. The five different colors represent each lineage (blue, A1; green, A2a; lime, A2b; red, B1; and pink, B2). The genes of JN184400 and KF516922 are colored deep green and black, respectively. Please see the Fig 1 legend for the information of isolation time points of sequences and posterior probability of tree branches.

We first determined the phylogenetic relationships of the complete genome and individual N, P, M, F, M2, SH, G, and L genes from 103 complete HMPV sequences (S2 and S3 Tables). In the MCC trees, the sequences of the complete and individual genes constituted the five different lineages of A1, A2a, A2b, B1, and B2, and the eight genes from each virus were all assigned to the same lineage (Figs 1–3 and S1 Fig).

There were, however, two exceptions to the lineage consistency. The first exception was a TN94-49 virus isolated in the U.S. in 1994 (TN94, GenBank accession number JN184400). The N, P, and F genes of TN94 were located in the A2a sublineage (Fig 1A, 1B and 1C), whereas its SH, G, and L genes were in the A2b sublineage (Fig 2A, 2B and 2C) and its M and M2 genes were at the A2 parental locations (Fig 3A and 3B). The second example was more divergent. The N, P, M, M2, SH, and L genes of the HMPV/AUS/144834728/2003/A virus (AUS03, GenBank accession number KC562241) were all grouped with other A1 lineage sequences (Figs 1–3); however, the F gene was located independently between the A1 and A2 lineages (Fig 1C), and the G was assigned to the A2b sublineage (Fig 2B).

**Fig 4:**
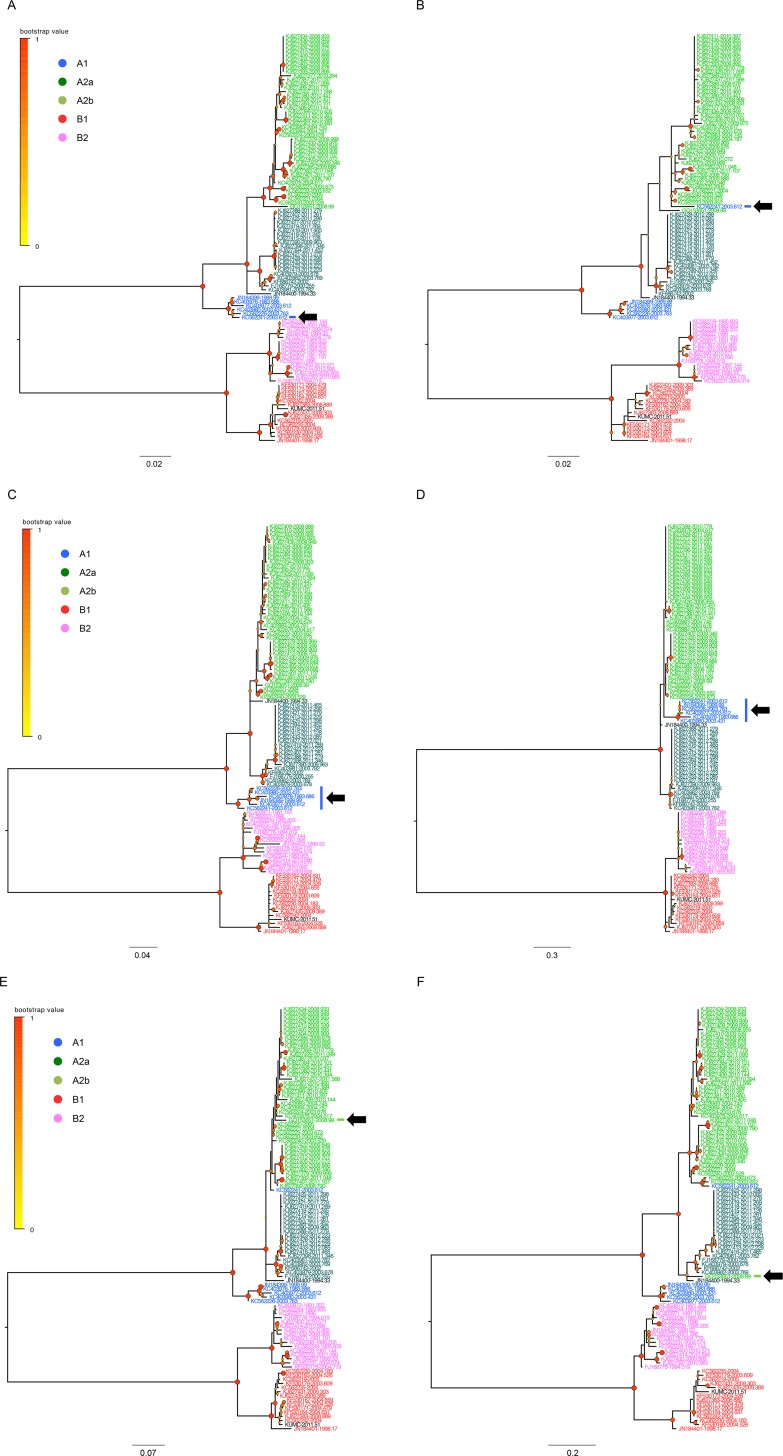
Phylogenetic placements of the pre- and post-breakpoint region sets of F, SH, and G gene sequences. The phylogenetic placements of the same viral sequences were compared using the datasets of pre- (A, C, and E) and post-breakpoint (B, D, and F) regions divided by the detected recombination breakpoints in the F (A and B), SH (C and D), and G (E and F) genes. The trees were reconstructed by MEGA5.2 and edited in the FigTree (v1.4). The scales in the trees indicate the number of substitutions per site.

As the genes of HMPVs are consistently positioned in a linear genome [1], phylogenetic discordance between the genes of an individual HMPV might suggest past recombination events [39]. To test this hypothesis, we analyzed the sequence sets (nucleotide and amino acid) of nine protein-coding regions (after the M2 gene sequences were separated into two protein-coding regions M2-1 and M2-2) using the SBP and GARD methods, which could detect potential recombination breakpoints [49]. We screened the nucleotide sequence sets first, and then, checked the corresponding amino acid sequence sets of nine coding regions. The GARD detected no breakpoints of recombination. However, the SBP method located three potential breakpoints: 1,207 nucleotide residue in the F gene, 153 amino acid residue in the SH gene, and 104 amino acid residue in the G gene (Table 1). To confirm these, we divided each sequence set into pre- and post-breakpoint regions and checked the placements of the same viral sequences in the phylogenetic trees (Fig 4). For the F and G genes, we found that some strains showed different phylogenetic locations (S4 Table). The pre- and post-breakpoint sequences of the F gene of AUS03 virus was located in the A1 and A2b lineages (Fig 4A and 4B), respectively, while those of the G gene of HMPVgz01 (GenBank accession number GQ153651) were placed in the A2b and A2a, respectively. For the G gene of TN94 (GenBank accession number JN184400), we also observed its different locations in the pre- (parental of A2a and A2b sublineages) and post-breakpoint (A2a sublineage) trees (Fig 4E and 4F). A drastic change between the lineages was observed in the pre- and post-breakpoint trees of the SH gene. The pre-breakpoint region of the A1 SH sequences was placed in the same ancestral location in relation to those of the A2 lineage (Fig 4C), as seen in other HMPV trees (Figs 1–3). However, the post-breakpoint region of the A1 SH sequences appeared to have a more close evolutionary relationship to the A2b sequences by being located between the A2a and A2b sublineages (Fig 4D and S4 Table). When the detected recombination candidates were removed from the sequence sets, there was no more recombination breakpoint detected in the F gene, but the same residues were still detected in the SH and G genes which suggesting a possible remainder of intra-lineage recombination imprints (S5 Table). The complete genome sequences also resulted in a similar result of recombination (Table 1 and S2 Fig). Considered together, these results indicate that genetic recombination within and between the HMPV lineages might have affected the evolutionary dynamics of HMPV genomes.

**Table 1:** Potential recombination breakpoints in the HMPV genomes.

	Potential breakpoint (BP)		Nucleotide region	
Coding region	Nucleotide	Amino acid	Pre-BP	Post-BP
Complete	4,308 (6773.75, 100%)^a^	n.d.^b^	1–4,305	4,309–12,180
F	1,207 (16.60, 99.99%)	n.d.	1–1,206	1,210–1,617
SH	n.d.	153 (2327.67, 100%)	1–456	460–531
G	n.d.	104 (4528.41, 100%)	1–309	313–444

^a^ IC improvement score and % model-averaged support based on the Akaike information criterion (AIC).

^b^ n.d., not detected.

### Selection profiles of HMPV genomes

Understanding the effects of selection pressures on the genetic diversity is of importance to interpret the evolutionary dynamics of HMPVs [54]. To address these, we analyzed overall dN/dS ratios of the complete and individual genes of HMPVs, and the SH and G genes exhibited higher dN/dS estimates than others. The three lowest dN/dS ratios were found in the N, M, and F genes. By the SLAC method, only the three codons (113, 127, and 139) in the G gene appeared to have been under positive selection whereas most others have been affected by negative selection to varying degrees (N, 13.28%; P, 13.56%; M, 11.41%; F, 12.43%; M2-1, 12.30%; M2-2, 2.82%; SH, 7.34%; G, 8.78%; and L, 9.61%) (Table 2). It is difficult to estimate positive selection using the sequence alignment of protein coding regions because most selection events might be episodic that happened only in some evolutionary lineages. To reduce this methodological limitation, the MEME method was suggested for the detection of both pervasive and episodic histories of positive selection [53], and it detected such sites in the N (1 site, 0.26% selection ratio), P (2, 0.68%), M (1, 0.39%), M2-1 (1, 0.53%), SH (3, 1.69%), G (5, 3.38%), and L (11, 0.54%) coding regions (Table 2 and S6 Table). However, the F and M2-2 coding regions exhibited no traces of positive selection, again. For the three surface glycoprotein genes (F, SH, and G), we further investigated the positive selection profiles of the five genetic lineages, and they mostly exhibited lineage-specific selection, compared with the results of overall gene sequences (S6 Table). The complete genome sequence set produced somewhat different positive selection codons, but for the G protein gene, it included all of the codons that were detected in the individual gene analysis (S6 and S7 Tables). These all suggest that not only negative selection but also pervasive and episodic events of positive selection interact to shape the genetic diversity of HMPVs.

**Table 2:** Selection profiles estimated from the HMPV genomes.

			Number of selected codons		
			Positive		Negative
Coding region	Codon #	Overall dN/dS	SLAC	MEME	SLAC
Complete	4,060	0.098	6 (0.15)^a^	54 (1.33)	798 (19.66)
N	384	0.035	n.d.^b^	1 (0.26)	51 (13.28)
P	295	0.120	n.d.	2 (0.68)	40 (13.56)
M	254	0.026	n.d.	1 (0.39)	29 (11.41)
F	539	0.045	n.d.	n.d.	67 (12.43)
M2-1	187	0.073	n.d.	1 (0.53)	23 (12.30)
M2-2	71	0.160	n.d.	n.d.	2 (2.82)
SH	177	0.380	n.d.	3 (1.69)	13 (7.34)
G	148	0.517	3 (2.03)	5 (3.38)	13 (8.78)
L	2,005	0.053	n.d.	11 (0.54)	197 (9.61)

^a^ The selection ratio (%) is provided in parenthesis.

^b^ n.d., not detected.

### Evolutionary profiles of HMPV genomes

The evolutionary rate of the complete genome of HMPVs was approximately 0.52 × 10^−3^ substitution/site/year (95% highest probability density (HPD) 0.27–0.76). The two membrane glycoprotein genes, SH and G, showed higher evolutionary rates (1.57 and 2.13 × 10^−3^ substitution/site/year, respectively) whereas those of the nucleocapsid and polymerase complex protein genes were found to be relatively low: N, 0.65 × 10^−3^ (0.36–0.92 × 10^−3^); P, 0.85 (0.42–1.29); M, 0.76 (0.38–1.16); M2, 0.74 (0.42–1.06); and L, 0.43 (0.19–0.63) (Table 3). However, another membrane glycoprotein gene F exhibited only 0.60 × 10^−3^ (0.32–0.90) evolutionary rate, which was the second lowest among all of the tested genes. At the translated amino acid level, the F protein gene again showed the lowest evolutionary rate (non-synonymous substitution), along with other N, M, and L protein genes, at less than 1 × 10^−5^ substitution/site/year. The G protein gene exhibited the highest evolutionary rate of amino acid at 1.10 × 10^−3^ substitution/site/year, followed by SH, P, M2-2, and M2-1, with 0.61, 0.20, 0.19, and 0.11 × 10^−3^ substitution/site/year, respectively (Table 3).

**Table 3:** Evolutionary rates estimated from the HMPV genomes.

		Evolutionary rate (10^−3^ substitutions/site/year)		
Coding region	NT length analyzed	Nucleotide	Synonymous	Nonsynonymous
Complete	12,133	0.52 (0.27–0.76) ^a^	-	-
N	1,152	0.65 (0.36–0.92)	0.52	< 10^−5^
P	885	0.85 (0.42–1.29)	0.50	0.20
M	762	0.76 (0.38–1.16)	0.55	< 10^−5^
F	1,617	0.60 (0.32–0.90)	0.43	< 10^−5^
M2	727	0.74 (0.42–1.06)	-	-
M2-1	564	-	0.62	0.11
M2-2	216	-	0.53	0.19
SH	531	1.57 (0.71–2.49)	0.57	0.61
G	444	2.13 (1.10–3.18)	0.72	1.10
L	6,015	0.43 (0.19–0.63)	0.32	< 10^−5^

^a^ Lower and upper limits of 95% HPD are provided in parenthesis.

**Fig 5:**
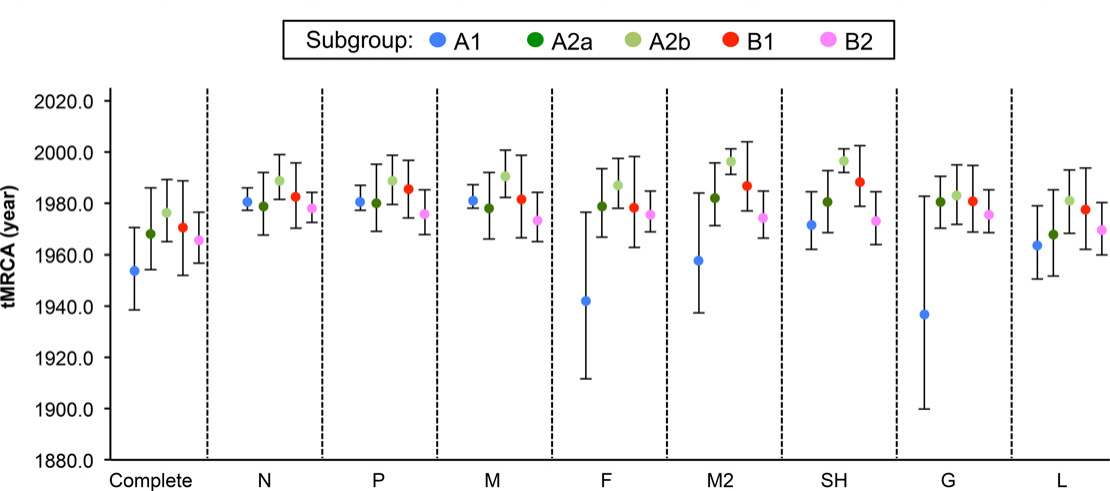
Time of the most recent common ancestor (tMRCA) of HMPV gene sequences. Based on a Bayesian analysis method implemented in BEAST v1.8.1, the estimated tMRCA of the eight HMPV genes is represented for each lineage. The lower and upper limits correspond to the 95% highest probability density (HPD).

We also estimated the circulation times of the common ancestors of the HMPV genes. The tMRCA of complete genome sequences of HMPVs was estimated as approximately 390.92 years of age, based on the most recent sequence age (2012.30 in year fraction), and that of the eight genes was also quite remote, ranging from 224.03 to 472.45 (N, 255.01; P, 288.46; M, 254.42; F, 289.30; M2, 224.03; SH, 255.70; G, 352.79; and L, 472.45) (S8 Table). However, the tMRCAs of each individual lineage were of recent origin (less than 50 years), except for the F, M2, and G genes in the A1 lineage, which were estimated to be 70.19 (95% HPD, 39.73–104.60), 54.56 (34.20–80.81), and 75.64 (38.83–121.64) years, respectively. Compared with other lineages, the A2b sequences appeared to have the most recent tMRCAs (15.88 to 31.45 years) (Fig 5). These suggest that the nucleocapsid and polymerase complex protein genes along with the surface glycoprotein F gene are less dynamic in molecular evolutionary rates than two other surface glycoprotein SH and G genes and that the tMRCA estimates might be indicative of divergent genetic distances between the individual lineages of HMPVs.

### Skyline-plot reconstruction and genetic diversity of HMPVs

**Fig 6:**
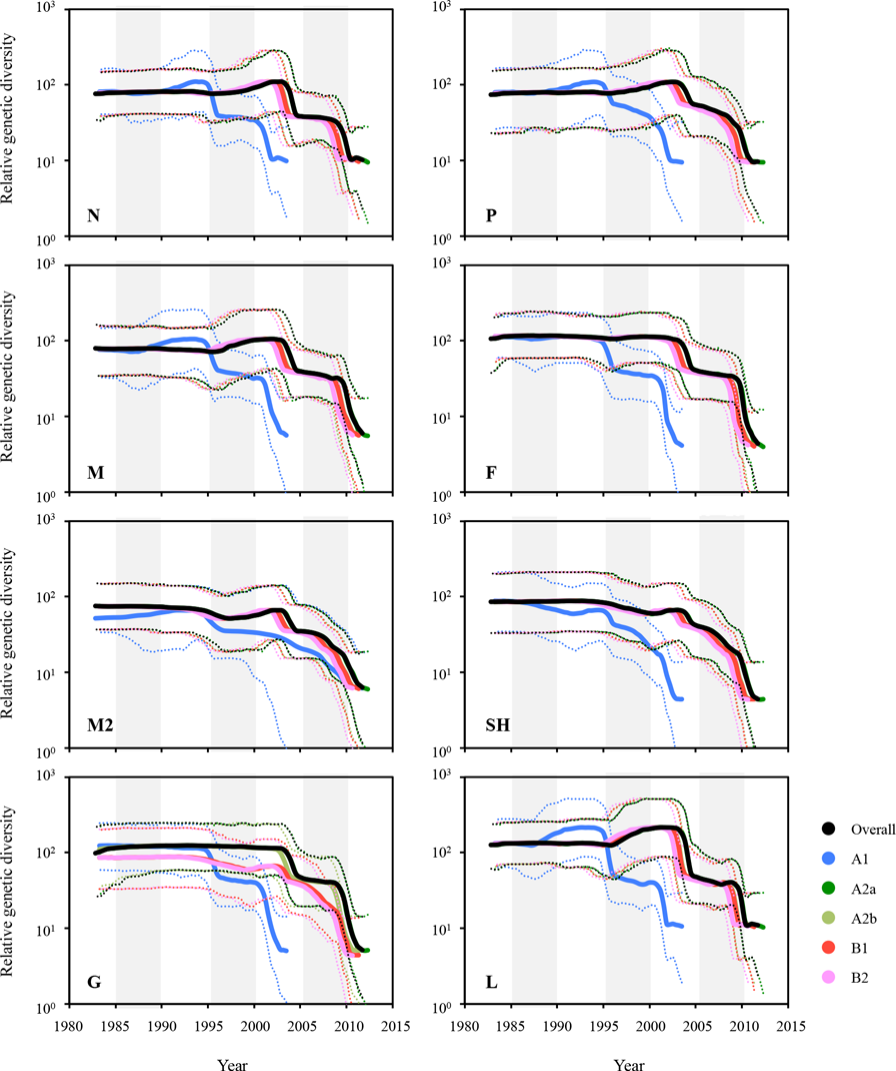
Population dynamics of HMPV sequences in skyline reconstruction plots. The demographic inferences of HMPV genetic diversity were depicted using a Bayesian skyline reconstruction plot method. The plots for the eight genes were then reconstituted into the five lineages. Solid lines represent mean diversity, and dotted lines represent the lower and upper limits of the 95% HPD.

The demographic legacy of HMPV genetic diversity was inferred using a Bayesian skyline-plot reconstruction method [25, 39]. It was revealed that the genetic diversity of the HMPV genes ranged from 4.38 to 215.79 (S7 Table). The A2a, A2b, B1, and B2 lineages of the N, P, M, and L genes showed the increases of genetic diversity between 2000 and 2005, whereas the A1 lineage of these genes displayed a similar peak in rather earlier times (approximately 1990–1995). Unlike the results of dN/dS ratio and evolutionary rates (Tables 2 and 3), the L gene showed the highest genetic diversity among all of the tested genes (Fig 6 and S9 Table). Among the three surface glycoprotein genes, the G gene exhibited the highest genetic diversity, and the A lineages of G gene exhibited more divergent demographic patterns than the B lineages whereas the two other glycoprotein genes, F and SH, showed similar patterns of diversity between the lineage lineages (Fig 6 and S9 Table). The genetic diversity of complete genome sequences appeared to be relatively higher, which might be mostly affected by that of the L gene due to its largest genome size among the eight genes of HMPVs (S9 Table).

### Phylogenetic location and genetic similarity of KUMC-MP virus genes

A KUMC-MP virus (GenBank accession number KF516922) was isolated from a nasopharyngeal aspirate specimen collected from an infant (eight months old, male) who had recovered from a lower respiratory infection and meningitis. In the MCC trees, eight genes of the KUMC-MP virus were all classified with the other B1 lineage sequences according to their phylogenetic relationships (Figs 1–3). More than 97.7% genetic similarity was found with the viruses from Australia and the U.S. isolated around 2004. Against the A lineage sequences, however, the SH and G genes of the KUMC-MP virus exhibited only 46.8% and 17% similarity, respectively (SH, KJ627401, isolated in 2011; and G, KJ627399, isolated in 2010), and the six other genes of KUMC-MP showed 72.4–81.8% similarity (Table 4).

**Table 4:** Sequence identity of KUMC-MP genes compared with other HMPV sequences.

	% similarity to KUMC-MP gene							
Classification	N	P	M	F	M2	SH	G	L
Overall	90.7	87.1	90.4	89.7	90.7	75.5	61.3	90
Maximum	99.3	98.5	99.9	99.3	98.7	97.9	97.7	99.1
	KC562242	KC562242	KF530164	KF530164	KC562242	KC562230	KF530164	KF530164
		KF530164	KF530167	KF530167	KF530163	KC562242		KF530171
		KF530171	KF530171	KF530171		KF530171		
			KF530173	KF530173				
	(B1/USA)	(B1/AUS^a^, USA)	(B1/AUS)	(B1/AUS)	(B1/AUS, USA)	(B1/AUS, USA)	(B1/AUS)	(B1/AUS)
Minimum	81.8	72.4	80.6	79.1	80.3	46.8	17	79.6
	KJ627405	JN184400	KC403981	KJ627381	KC403977	KJ627401	KJ627399	JN184399
				KJ627389				KC403980
				KJ627396				KC562226
				KJ627411				KC562241
				KJ627437				
	(A2b/Peru)	(A2a/USA)	(A2a/AUS)	(A2b/Peru)	(A1/AUS)	(A2b/Peru)	(A2b/Peru)	(A1/AUS, USA)

^a^ AUS, Australia.

## Discussion

Most previous studies evaluating the molecular evolution of HMPVs utilized a few genes instead of using a complete genome set. By great help of two recent genomic sequencing projects of HMPVs (BioProject PRJNA73051 and 237298), we could, for the first time, investigate the evolutionary footprints of the eight, complete genes of 103 HMPVs. However, most sequences were obtained from Peru (n = 57), Australia (n = 23), and USA (n = 18) regions (S2 Table). This might bias the results we observed even though the five genetic lineages of HMPVs were all present in the phylogenetic trees (Figs 1–3) and no certain lineages dominated in these geographical regions.

Given the individual tMRCAs, each lineage of HMPVs appeared to be relatively recent origins (Fig 5 and S6 Table). However, when all of the lineage sequences were considered together, the overall tMRCAs extremely increased to the ranges of 224.03 to 472.45 years (S8 Table). Although a previous report also mentioned remote tMRCAs of some genes of HMPVs [25], the tMRCAs presented in this study provided comprehensive information of the eight genes with regard to the times of their undetected circulation and their relation to the inter-lineage diversity of HMPVs. In fact, the higher tMRCAs of HMPV genes were consistently matched with the higher mean values of genetic diversity, except for the N and SH genes (S8 and S9 Tables). These might suggest that the lineages of HMPVs experience ongoing evolution of genetic divergence and propose a question of what forces affect the evolutionary dynamics of HMPVs. We suspected genetic recombination first. In the MCC trees, we observed that the genes of TN94 and AUS03 viruses exhibited phylogenetic discordance (Figs 1–3). Given that HMPVs have a linear, single-stranded RNA genome, phylogenetic congruence is expected among the eight genes of the same virus. However, the TN94 virus harbored A2/A2a/A2b-lineage genes, and the AUS03 virus carried A1/A2/A2b-lineage genes. We speculated that past recombination events might have caused these results, as was suspected in a previous study [39]. To test this hypothesis, we examined the complete and individual sequence sets using the two different methods, SBP and GARD. When evaluating the nucleotide sequence sets by the SBP method, only the F gene appeared to retain one potential breakpoint of recombination. For the corresponding amino acids, the SH and G were also detected by the same method (Table 1). Different sensitivity to recombination analysis between nucleotide and amino acid sequences cannot be fully understood here, but it might be resulted from the various magnitudes of signal-to-noise ratios retained in our sequence sets because nucleotide sequence sets might have more obscure genetic relationships that should be taken into account for phylogenetic reconstruction, compared with amino acid sequence sets [55]. Genetic diversity retained in the sequence sets might be another reason for different sensitivity to recombination analysis. However, it might be still speculative because none was evidenced by the second method GARD (Table 1). These might be then spurious, so we tried to confirm the results by checking the lineage placements of the same viral gene sequences in the trees that were reconstructed using the sequence sets of pre- and post-breakpoint regions. One or two different viral sequences per gene exhibited a change of lineages in the phylogenetic trees of F and G genes (Fig 4A, 4B, 4E and 4F). For the SH gene, the pre- and post-breakpoint regions of the A1 lineage sequences all appeared to have different phylogenetic relationships to those of the A2a and A2b lineage sequences (Fig 4C and 4D). The different relationship of the same viral sequences in the phylogenetic trees indirectly indicated that previous recombination events were imprinted in the HMPV genomes and demonstrated that the inter-lineage interaction due to recombination might affect the genetic diversity of HMPVs. Also, the intra-lineage imprints of recombination could be another factor that worked on the HMPV genomes because the SH and G genes still retained the same recombination imprints even after the removal of the sequences of phylogenetic incongruences between the pre- and post-breakpoints trees (S5 Table). When analyzed with the complete genome sequences, a similar result was also obtained, and it was confirmed again in the different phylogenetic placements between the pre- and post-breakpoint sequence sets (S2 Fig and S4 Table). Especially for the KC562241 strain, the two post-breakpoint sets of the complete genome sequences failed to demonstrate its potential as a recombinant strain (S2B Fig). However, it was confirmed when removing the L gene region from the post-breakpoint sequence set because of its largest gene size (S2C Fig). The possibility of false negative in the GARD should be also counted because the genetic diversity reflected in the aligned sequences could be below the limit required for the detection of recombination, or an unequal distribution of time intervals across the intra- and inter-lineages might have distorted the results. Artifacts caused by some technical errors, such as plasmid contamination during sequence reading procedures or co-infection possibility of two different HMPVs in a targeted clinical specimen, should be also considered.

Recombination is considered to contribute to the genetic diversity of viruses [56, 57]. Through this genomic interaction, a virus may evade host immunity, expand its host range, or generate a totally new virus strain [58]. Given that the F genes of HMPVs appear to be relatively conserved across different lineages [59] and that the *Pneumovirinae* share a common fusion mechanism [1], recombination among the F genes might contribute to their genomic preservation. As observed in an example of the removal of deleterious mutations [60], the HMPV F protein might maintain its fusion activity through recombination process. In this case, natural selection might be another evolutionary force acting on the bottleneck clones [61]. Negative selection has important implications for RNA-virus evolution because a large number of rare nonsynonymous mutations resulting from viral replication are often purified from the population [62]. It was previously provided that the HMPV genes were affected by negative selection pressure [32]. As presented in Table 2, we also observed that most of the genes were affected by negative selection. Along with purifying, negative selection pressure, episodic diversifying selection could be another evolutionary force working on the HMPV genomes. Even though only three sites in G protein genes were positively selected by the SLAC method, the MEME method located the imprints of both pervasive and episodic diversifying selections in the seven protein coding regions (Table 2), and this was demonstrated again in the results of the complete and separate lineage sequences (S6 and S7 Tables). Especially, the residue 146 in the G gene was suggested as one of the positive selection codons among the A2a sublineage sequences, and it was also detected in the results of the de Graaf et al. [25] and Papenburg et al. [18] studies. This might indicate the importance of this residue for the adaptive evolution of HMPVs. However, these natural selection profiles should be interpreted with care due to the suspected past recombination events. Or, these all may be tested using mutant viruses. By using the reverse genetically-rescued viruses, the implications of recombination and natural selection acting on some target residues can be further elucidated as demonstrated in other RNA viruses [63, 64].

The demographic distribution of the effective population size is indicative of genetic diversity [58]. As reported previously [25], most of the HMPV genes showed relatively low genetic diversity, except for the L gene (Fig 6 and S7 Table). The L gene sequences showed the lowest evolutionary rates in both the nucleotide and amino acid estimates (Table 3). In the selection profiles, the L gene also exhibited a low dN/dS ratio and 9.61% negative selection (Table 2). Considering that the L gene encodes a viral RNA polymerase and that a larger genome is usually associated with a lower mutation rate, the low rates of substitution and dN/dS values found for the HMPV L gene are understandable. However, the L gene showed the most increased diversity during 2000–2005, compared with the other genes (Fig 6 and S9 Table). This difference should be further investigated to examine the effects of L gene diversity on HMPV evolution. The G gene also exhibited interesting demographic pattern. As seen in Fig 6, the five lineages of HMPVs shared very similar demographic distributions even though the A1 lineage always showed its demography at an earlier time period. However, the A lineages of G gene exhibited a higher genetic diversity than the B lineages. This lineage-dependent genetic diversity should be also addressed in the near future to better understand the molecular dynamics of HMPV evolution. In addition to the relatively conserved F gene, which considered the best candidate vaccine antigen against HMPV infection [59, 65], the genetically divergent characteristics of G genes across different genetic lineages may provide information that can be applied into the development of lineage-specific or multivalent HMPV vaccines and antivirals [11].

We isolated the KUMC-MP virus from a clinical sample collected in 2011. Based on the analysis of genetic similarity, we suspected this virus might be genetically related with the B1 lineage viruses circulating in Australia or the U.S. around 2004 (Table 4). Given the sustained circulation of HMPVs in Korea [66, 67], more genomic data are needed to investigate the molecular epidemiology and evolutionary dynamics of HMPVs in Korea.

In conclusion, we investigated the evolutionary dynamics of HMPVs by analyzing the complete genome sequences. Based on our results, we suggest that recombination is one of the evolutionary forces to have affected the genetic diversity within and between the five genetic lineages of HMPVs and that not only negative selection but also episodic positive selection works on shaping the evolutionary structure of HMPVs.

## Supporting Information

S1 Data Data set of 103 HMPV genome sequences used in this study. (ZIP)

S1 Fig Phylogenetic relationship of the complete genome sequences of HMPVs. The relative phylogenetic relationship of complete genome sequences of HMPVs was defined in time-framed maximum clade credibility (MCC) trees. The five different colors represent each lineage (blue, A1; green, A2a; lime, A2b; red, B1; and pink, B2). The genes of JN184400 and KF516922 are colored deep green and black, respectively. The isolation time point of each sequence is expressed as the year fraction at the end of the sequence accession number. As the color of circles in the tree nodes, the size of circles in the node represents the posterior probability of their clustering (the bigger size demonstrates the higher probability).
(TIF)

S2 Fig Comparison of phylogenetic placements between the pre- and post-breakpoint region sets of the complete genome sequences of HMPVs. The phylogenetic placements of the same viral sequences were compared using the datasets of pre- (A, using 1–4,305 nucleotide region) and post-breakpoint (B, using 4,309–12,180 nucleotide region and C, using 4,309–6,165 nucleotide region) regions divided by the detected recombination breakpoint (4,305 in nucleotide) in Table 1. The trees were reconstructed by MEGA5.2 and edited in the FigTree (v1.4). The scale in the trees indicate the number of substitutions per site.
(TIF)

S1 Table Primers used for reverse transcription-PCR and DNA sequencing. (DOCX)

S2 Table Subtype and geographic information of complete HMPV sequences. (DOCX)

S3 Table Information of lineage, strain name, isolation region and year of HMPVs. (DOCX)

S4 Table Putative recombination strains based on the phylogenetic placements between the pre- and post-breakpoint sequence sets in Fig 4. (DOCX)

S5 Table Recombination breakpoints detected after removing putative recombination strains. (DOCX)

S6 Table Positive selection codons estimated from the HMPV genomes. (DOCX)

S7 Table Positive selection codons estimated from the complete genome sequences of HMPVs. (DOCX)

S8 Table Estimated time of most recent common ancestors (tMRCA) of the HMPV genomes. (DOCX)

S9 Table Index of relative genetic diversity of the HMPV genomes. (DOCX)
