# Supplementary figures and images for: Genome-Wide Analysis of Human Metapneumovirus Evolution
Source: PLoS One. 2016 Apr 5;11(4):e0152962. doi: 10.1371/journal.pone.0152962 (PMC4821609; doi:10.1371/journal.pone.0152962)

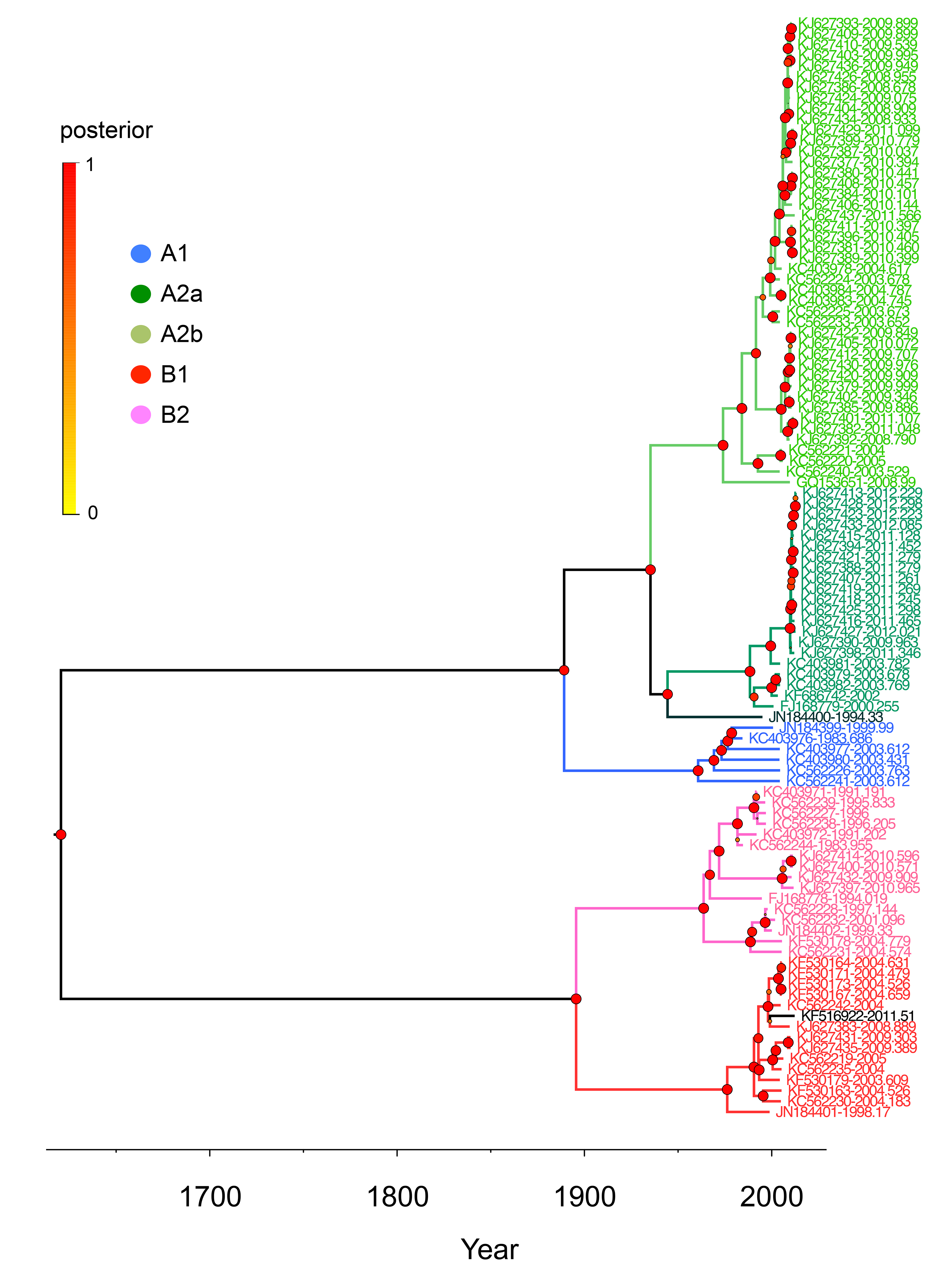

Supplement: S1 Fig — The relative phylogenetic relationship of complete genome sequences of HMPVs was defined in time-framed maximum clade credibility (MCC) trees. The five different colors represent each lineage (blue, A1; green, A2a; lime, A2b; red, B1; and pink, B2). The genes of JN184400 and KF516922 are colored deep green and black, respectively. The isolation time point of each sequence is expressed as the year fraction at the end of the sequence accession number. As the color of circles in the tree nodes, the size of circles in the node represents the posterior probability of their clustering (the bigger size demonstrates the higher probability). (TIF) [file pone.0152962.s002.tif]

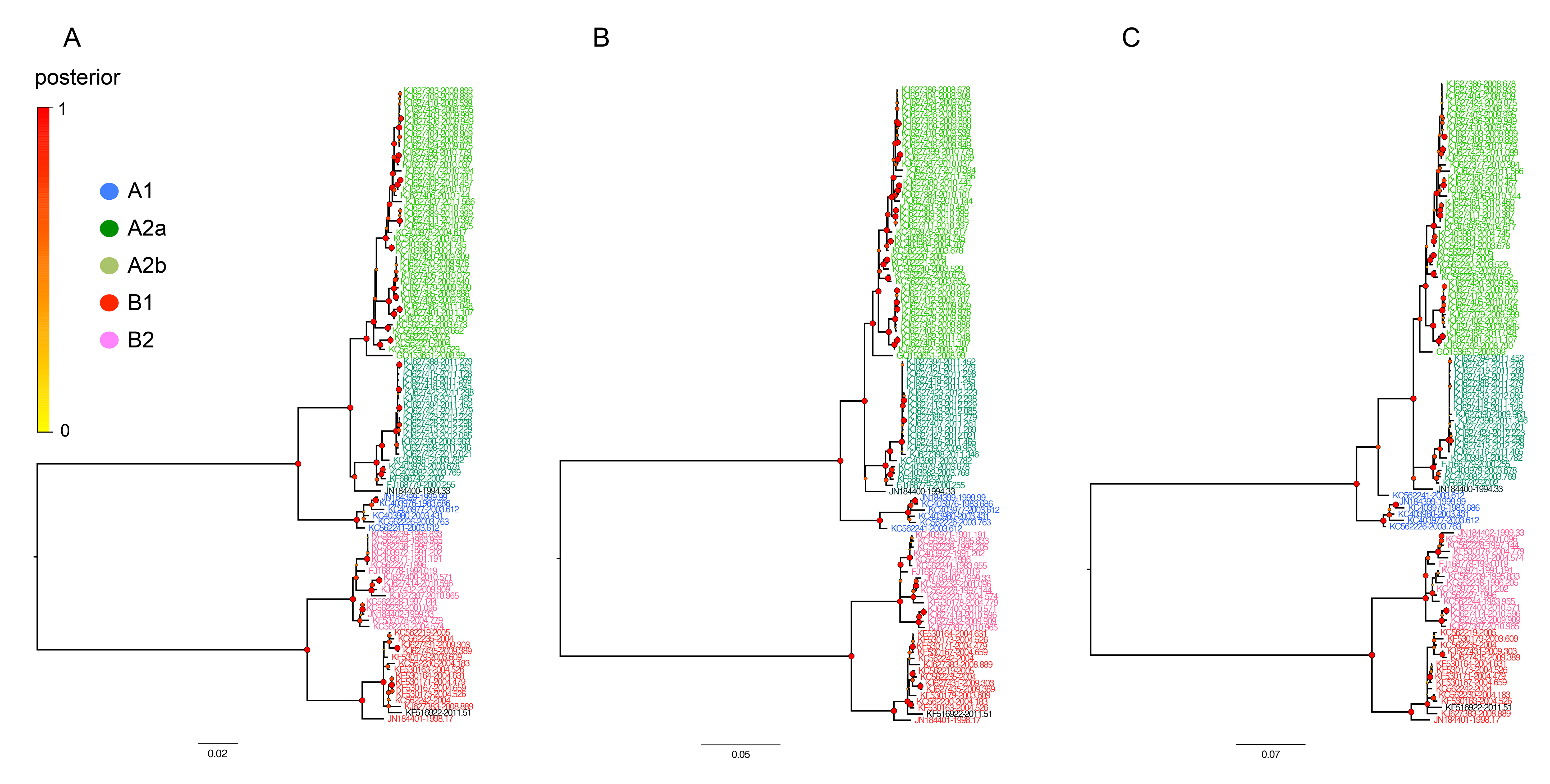

Supplement: S2 Fig — The phylogenetic placements of the same viral sequences were compared using the datasets of pre- (A, using 1–4,305 nucleotide region) and post-breakpoint (B, using 4,309–12,180 nucleotide region and C, using 4,309–6,165 nucleotide region) regions divided by the detected recombination breakpoint (4,305 in nucleotide) in Table 1. The trees were reconstructed by MEGA5.2 and edited in the FigTree (v1.4). The scale in the trees indicate the number of substitutions per site. (TIF) [file pone.0152962.s003.tif]
