# Supplementary material for: Genome-Wide Analysis of Human Metapneumovirus Evolution
Source: PLoS One. 2016 Apr 5;11(4):e0152962. doi: 10.1371/journal.pone.0152962 (PMC4821609; doi:10.1371/journal.pone.0152962)
Supplement: S1 Table — (DOCX) [file pone.0152962.s004.docx]

**S1 Table. Primers used for reverse transcription-PCR and DNA sequencing.**

| - - - 1. Primer | - - - 1. Sequence (5’3’) | - - - 1. Size (nt) | - - - 1. Region^a^ | - - - 1. Reference |
| --- | --- | --- | --- | --- |
| - - - 1. Random Hexamer | - - - 1. NNN NNN | - - - 1. 6 |  | - - - 1. - |
| - - - 1. Meta-M-OF2 | - - - 1. CAATATGGTTCCCTTTGTTTCAGGCCA | - - - 1. 27 | - - - 1. 2223-2249 | - - - 1. a |
| - - - 1. Meta-M-OR2 | - - - 1. TGGTCTGCTTCACTGCTTATWGCAGCTT | - - - 1. 28 | - - - 1. 2659-2686 |  |
| - - - 1. Meta-M-IF2 | - - - 1. CCCTTTGTTTCAGGCCAAYACACCACC | - - - 1. 27 | - - - 1. 2234-2260 |  |
| - - - 1. Meta-M-IR2 | - - - 1. GCAGCTTCAACAGTRGCTGATTCACTCTC | - - - 1. 29 | - - - 1. 2689-2717 |  |
| - - - 1. NCR-1F | - - - 1. ACGCGAAAAAAACGCGTATA | - - - 1. 20 | - - - 1. NCR 1-20 | - - - 1. - |
| - - - 1. N-1F | - - - 1. ATGTCTCTTCAAGGGATTCA | - - - 1. 20 | - - - 1. 1-20 |  |
| - - - 1. N-87F | - - - 1. AGGCACCACAACTGCAGTGACA | - - - 1. 22 | - - - 1. 87-108 |  |
| - - - 1. N-937R | - - - 1. ACAGCCCTGCCTTTGGACTTTGT | - - - 1. 23 | - - - 1. 915-937 |  |
| - - - 1. NP-1384R | - - - 1. GCGGGTTTGCTGATGGTGGGT | - - - 1. 21 | - - - 1. 1365-1384 |  |
| - - - 1. P-1552R | - - - 1. GCGGGAGTCTTCCCATCACTGGA | - - - 1. 22 | - - - 1. 1531-1552 |  |
| - - - 1. P-1374F | - - - 1. CCCACCATCAGCAAACCCGCAC | - - - 1. 22 | - - - 1. 1374-1395 |  |
| - - - 1. P-1425F | - - - 1. GCATGGGCAGACAACAGCGGA | - - - 1. 21 | - - - 1. 1425-1445 |  |
| - - - 1. M-2239F | - - - 1. TATTTCAGGCCAACACACCA | - - - 1. 20 | - - - 1. 2239-2258 |  |
| - - - 1. M-2437F | - - - 1. TTGACAAGCTGACGGT | - - - 1. 16 | - - - 1. 2437-2452 |  |
| - - - 1. PM-2694R | - - - 1. TCAAGGCTTGGTCGGCTTCGC | - - - 1. 21 | - - - 1. 2674-2694 |  |
| - - - 1. PM-2864R | - - - 1. TCCTTGGTGACTCCAGCTCT | - - - 1. 20 | - - - 1. 2845-2864 |  |
| - - - 1. F-3054F | - - - 1. ACCCCAGCACGGGCTAAAGGA | - - - 1. 21 | - - - 1. 3054-3074 |  |
| - - - 1. MF-3153R | - - - 1. AGACATTAGTGTACCAGCCTGTTC | - - - 1. 24 | - - - 1. 3130-3153 |  |
| - - - 1. MF-3888R | - - - 1. TCGGCAATTGAACCATGTAA | - - - 1. 20 | - - - 1. 3869-3888 |  |
| - - - 1. F-4144F | - - - 1. CTCGGTGCTTTGGTGGCTTGC | - - - 1. 21 | - - - 1. 4144-4164 |  |
| - - - 1. F-4421F | - - - 1. TCAGGCACTGGTGGAACAGTCA | - - - 1. 22 | - - - 1. 4421-4442 |  |
| - - - 1. F-4443R | - - - 1. TGACTGTTCCACCAGTGCCTGAC | - - - 1. 23 | - - - 1. 4421-4443 |  |
| - - - 1. F-4587R | - - - 1. CTGGAGGTGCTCCTGTGGGC | - - - 1. 20 | - - - 1. 4568-4587 |  |
| - - - 1. M2-1-5008F | - - - 1. GCTTTCTGATAGCAAACATGTGGCG | - - - 1. 19 | - - - 1. 5008-5026 |  |
| - - - 1. M2-1-5155F | - - - 1. AGCAGGAACTGACAATGACCCTTCA | - - - 1. 19 | - - - 1. 5155-5173 |  |
| - - - 1. M2-1-5290R | - - - 1. TTTACTATCCCATCAGATAGTGTTTC | - - - 1. 20 | - - - 1. 5266-5290 |  |
| - - - 1. SH-5916F | - - - 1. CTACATATAACCGAGTGGGGGTGCT | - - - 1. 25 | - - - 1. 5916-5940 |  |
| - - - 1. SH-5940R | - - - 1. AGCACCCCCACTCGGTTATATGT | - - - 1. 23 | - - - 1. 5918-5940 |  |
| - - - 1. G-6045F | - - - 1. CTAGGCTGCATGACACTTCGCA | - - - 1. 22 | - - - 1. 6045-6066 |  |
| - - - 1. G-6404F | - - - 1. CACAGCAGGCCCAAGCACCA | - - - 1. 20 | - - - 1. 6404-6423 |  |
| - - - 1. G-6784F | - - - 1. GCGAACAGACAACCCGGGCA | - - - 1. 20 | - - - 1. 6784-6803 |  |
| - - - 1. G-6804R | - - - 1. TGCCCGGGTTGTCTGTTCGC | - - - 1. 20 | - - - 1. 6785-6804 |  |
| - - - 1. L-7521F | - - - 1. TTATAGATGTGGAGTTCATACCYG | - - - 1. 24 | - - - 1. 7521-7544 |  |
| - - - 1. GL-7695R | - - - 1. TTACTACACATCCATAAGATGATA | - - - 1. 24 | - - - 1. 7676-7695 |  |
| - - - 1. L-7908F | - - - 1. TGCAATGAAGGATTCTCTCT | - - - 1. 20 | - - - 1. 7908-7927 |  |
| - - - 1. L-8101F | - - - 1. CCCTATGTAYGAAGTAGTACTTAAA | - - - 1. 25 | - - - 1. 8101-8125 |  |
| - - - 1. L-8253R | - - - 1. GCATCCATTGCTTCCCTCTCATC | - - - 1. 23 | - - - 1. 8231-8253 |  |
| - - - 1. L-8496R | - - - 1. TSCTCAAATTGYACTGCAGCAAGTTC | - - - 1. 26 | - - - 1. 8471-8496 |  |
| - - - 1. L-8521F | - - - 1. CAACCTTGAGATGGTATTAAATGAT | - - - 1. 25 | - - - 1. 8521-8545 |  |
| - - - 1. L-9005 F | - - - 1. GCAAGRGCCTCTATAGTAACAGACTT | - - - 1. 26 | - - - 1. 9005-9030 |  |
| - - - 1. L-9100 R | - - - 1. ATGTAACTCATCAGCTACATCTGCAC | - - - 1. 26 | - - - 1. 9075-9100 |  |
| - - - 1. L-9401F | - - - 1. GGTATAGATGAAGTAAAAGCAGAC | - - - 1. 24 | - - - 1. 9401-9424 |  |
| - - - 1. L-10108R | - - - 1. GCTTGTCGTTCTGATCCTAC | - - - 1. 20 | - - - 1. 10089-10108 |  |
| - - - 1. L-10266F | - - - 1. GAGTGCTCTATGAATCACTACCTTT | - - - 1. 25 | - - - 1. 10266-10290 |  |
| - - - 1. L-10581F | - - - 1. TGGATGTTGTGTATGCAACT | - - - 1. 20 | - - - 1. 10581-10600 |  |
| - - - 1. L-10704R | - - - 1. CTTGAGTGCTTGATCCTACC | - - - 1. 20 | - - - 1. 10685-10704 |  |
| - - - 1. L-10707R | - - - 1. TCTCTTGAGTGCTTGATCCT | - - - 1. 20 | - - - 1. 10688-10107 |  |
| - - - 1. L-11476F | - - - 1. GGATTTCAARGTATTTCTATGTGTA | - - - 1. 25 | - - - 1. 11476-11500 |  |
| - - - 1. L-11671F | - - - 1. TGTGAAATTCCTATCATTAGTAGG | - - - 1. 24 | - - - 1. 11671-11694 |  |
| - - - 1. L-12087F | - - - 1. TTACCATGGCAACACGTAAACAGGT | - - - 1. 25 | - - - 1. 12087-12111 |  |
| - - - 1. L-12050R | - - - 1. TGTAGTCTGAACTGGTATCATAAC | - - - 1. 24 | - - - 1. 12027-12050 |  |
| - - - 1. L-12405F | - - - 1. ATTGGGACTTGATACACAGA | - - - 1. 20 | - - - 1. 12405-12424 |  |
| - - - 1. L-12528F | - - - 1. GTAGAATCTGTACAGCTTATGGAA | - - - 1. 24 | - - - 1. 12528-12505 |  |
| - - - 1. L-12841R | - - - 1. CTATCAGGATTGAGAATACCTATA | - - - 1. 24 | - - - 1. 12818-12841 |  |
| - - - 1. L-13124R | - - - 1. TAATCAAGGTCACTGGGTATATGC | - - - 1. 24 | - - - 1. 13101-13124 |  |
| - - - 1. L-13280R | - - - 1. GTATACATTCAATTTCAATTTCTG | - - - 1. 24 | - - - 1. 13257-13280 |  |

- - - 1. ^a^ Lam WY, Yeung AC, Tang JW, Ip M, Chan EW, Hui M, et al. Rapid multiplex nested PCR for detection of respiratory viruses. Journal of clinical microbiology. 2007;45(11):3631-40. doi: 10.1128/JCM.00280-07. PubMed PMID: 17804659; PubMed Central PMCID: PMCPMC2168518.
