# Supplementary material for: Genome-Wide Analysis of Human Metapneumovirus Evolution
Source: PLoS One. 2016 Apr 5;11(4):e0152962. doi: 10.1371/journal.pone.0152962 (PMC4821609; doi:10.1371/journal.pone.0152962)
Supplement: S2 Table — (DOCX) [file pone.0152962.s005.docx]

**S2 Table. Subtype and geographic information of complete HMPV sequences.**

|  | The number of sequences | | | | | | | | | | | | | |
| --- | --- | --- | --- | --- | --- | --- | --- | --- | --- | --- | --- | --- | --- | --- |
|  | Subgroup | | | | | |  | Region^a^ | | | | | | |
| Year | A1 | A2a | A2b | B1 | B2 | Total |  | AG | AUS | China | NL | Peru | USA | Korea |
| Before 2008 | 6 | 6 | 9 | 11 | 12 | 44 |  | 1 | 23 | - | 2 | - | 18 | - |
| Since 2008 | 0 | 16 | 35 | 4 | 4 | 59 |  | - | - | 1 | - | 57 | - | 1 |

^a^Abbreviations used in the region: AG, Argentine; AUS, Australia; and NL, Netherlands.
