# Supplementary material for: Genome-Wide Analysis of Human Metapneumovirus Evolution
Source: PLoS One. 2016 Apr 5;11(4):e0152962. doi: 10.1371/journal.pone.0152962 (PMC4821609; doi:10.1371/journal.pone.0152962)
Supplement: S3 Table — (DOCX) [file pone.0152962.s006.docx]

**S3 Table. Information of lineage, strain name, isolation region and year of HMPVs.**

| Lineage | Accession # | Strain name | Region | Year |
| --- | --- | --- | --- | --- |
| A1 | JN184399 | HMPV/USA/TN96-12/1999/A | USA | 1999 |
| (n = 6) | KC403976 | HMPV/USA/TN-83-93/1983/A | USA | 1983 |
|  | KC403977 | HMPV/AUS/145371295/2003/A | Australia | 2003 |
|  | KC403980 | HMPV/AUS/138758999/2003/A | Australia | 2003 |
|  | KC562226 | HMPV/AUS/150229278/2003/A | Australia | 2003 |
|  | KC562241 | HMPV/AUS/144834728/2003/A | Australia | 2003 |
| Subgroup | Accession # | Strain name | Region | Year |
| A2a | FJ168779 | NL/00/17 | Netherlands | 2000 |
| (n = 22) | JN184400 | TN94-49 | USA | 1994 |
|  | KC403979 | HMPV/AUS/145570377/2003/A | Australia | 2003 |
|  | KC403981 | HMPV/AUS/146892777/2003/A | Australia | 2003 |
|  | KC403982 | HMPV/AUS/143003542/2003/A | Australia | 2003 |
|  | KF686742 | HMPV/ARG/107/2002/A | Argentina | 2002 |
|  | KJ627388 | HMPV/Homo sapiens/PER/CFI1296/2011/A | Peru | 2011 |
|  | KJ627390 | HMPV/Homo sapiens/PER/FLE7544/2009/A | Peru | 2009 |
|  | KJ627394 | HMPV/Homo sapiens/PER/FPP00610/2011/A | Peru | 2011 |
|  | KJ627398 | HMPV/Homo sapiens/PER/FPP00505/2011/A | Peru | 2011 |
|  | KJ627407 | HMPV/Homo sapiens/PER/CFI1288/2011/A | Peru | 2011 |
|  | KJ627413 | HMPV/Homo sapiens/PER/CFI1669/2012/A | Peru | 2012 |
|  | KJ627415 | HMPV/Homo sapiens/PER/FPI01306/2011/A | Peru | 2011 |
|  | KJ627416 | HMPV/Homo sapiens/PER/FPP00544/2011/A | Peru | 2011 |
|  | KJ627418 | HMPV/Homo sapiens/PER/FPP00366/2011/A | Peru | 2011 |
|  | KJ627419 | HMPV/Homo sapiens/PER/FPP00408/2011/A | Peru | 2011 |
|  | KJ627421 | HMPV/Homo sapiens/PER/FPP00416/2011/A | Peru | 2011 |
|  | KJ627423 | HMPV/Homo sapiens/PER/CFI1657/2012/A | Peru | 2012 |
|  | KJ627425 | HMPV/Homo sapiens/PER/CFI1303/2011/A | Peru | 2011 |
|  | KJ627427 | HMPV/Homo sapiens/PER/FPP01153/2012/A | Peru | 2012 |
|  | KJ627428 | HMPV/Homo sapiens/PER/CFI1717/2012/A | Peru | 2012 |
|  | KJ627433 | HMPV/Homo sapiens/PER/IPE00957/2012/A | Peru | 2012 |
| Subgroup | Accession # | Strain name | Region | Year |
| A2b | GQ153651 | HMPV gZ01 | China | 2008 |
| (n = 44) | KC403978 | HMPV/AUS/172832103/2004/A | Australia | 2004 |
|  | KC403983 | HMPV/AUS/172837900/2004/A | Australia | 2004 |
|  | KC403984 | HMPV/AUS/193951503/2004/A | Australia | 2004 |
|  | KC562220 | HMPV/USA/C1-718//2005/A | USA | 2005 |
|  | KC562221 | HMPV/USA/C1-446/2004/A | USA | 2004 |
|  | KC562224 | HMPV/AUS/136342137/2003/A | Australia | 2003 |
|  | KC562225 | HMPV/AUS/141196482/2003/A | Australia | 2003 |
|  | KC562233 | HMPV/AUS/133878351/2003/A | Australia | 2003 |
|  | KC562240 | HMPV/AUS/134249451/2003/A | Australia | 2003 |
|  | KJ627377 | HMPV/Homo sapiens/PER/CFI0320/2010/A | Peru | 2010 |
|  | KJ627379 | HMPV/Homo sapiens/PER/FLE7574/2009/A | Peru | 2009 |
|  | KJ627380 | HMPV/Homo sapiens/PER/CFI0333/2010/A | Peru | 2010 |
|  | KJ627381 | HMPV/Homo sapiens/PER/FLI1921/2010/A | Peru | 2010 |
|  | KJ627382 | HMPV/Homo sapiens/PER/CFI1212/2011/A | Peru | 2011 |
|  | KJ627384 | HMPV/Homo sapiens/PER/FLE8237/2010/A | Peru | 2010 |
|  | KJ627385 | HMPV/Homo sapiens/PER/FLE6360/2009/A | Peru | 2009 |
|  | KJ627386 | HMPV/Homo sapiens/PER/FLA4032/2008/A | Peru | 2008 |
|  | KJ627387 | HMPV/Homo sapiens/PER/FLE7671/2010/A | Peru | 2010 |
|  | KJ627389 | HMPV/Homo sapiens/PER/FLI1302/2010/A | Peru | 2010 |
|  | KJ627392 | HMPV/Homo sapiens/PER/FLA4579/2008/A | Peru | 2008 |
|  | KJ627393 | HMPV/Homo sapiens/PER/FLE7209/2009/A | Peru | 2009 |
|  | KJ627396 | HMPV/Homo sapiens/PER/FLI1305/2010/A | Peru | 2010 |
|  | KJ627399 | HMPV/Homo sapiens/PER/FLI4745/2010/A | Peru | 2010 |
|  | KJ627401 | HMPV/Homo sapiens/PER/FPI01165/2011/A | Peru | 2011 |
|  | KJ627402 | HMPV/Homo sapiens/PER/FLA6964/2009/A | Peru | 2009 |
|  | KJ627403 | HMPV/Homo sapiens/PER/FLE7570/2009/A | Peru | 2009 |
|  | KJ627404 | HMPV/Homo sapiens/PER/FLA4816/2008/B | Peru | 2008 |
|  | KJ627405 | HMPV/Homo sapiens/PER/FLE7850/2010/A | Peru | 2010 |
|  | KJ627406 | HMPV/Homo sapiens/PER/FLE8149/2010/A | Peru | 2010 |
|  | KJ627408 | HMPV/Homo sapiens/PER/CFI0350/2010/A | Peru | 2010 |
|  | KJ627409 | HMPV/Homo sapiens/PER/FLE7210/2009/A | Peru | 2009 |
|  | KJ627410 | HMPV/Homo sapiens/PER/FLE0586/2009/A | Peru | 2009 |
|  | KJ627411 | HMPV/Homo sapiens/PER/FLI1311/2010/A | Peru | 2010 |
|  | KJ627412 | HMPV/Homo sapiens/PER/FLE4458/2009/A | Peru | 2009 |
|  | KJ627420 | HMPV/Homo sapiens/PER/FLE7219/2009/A | Peru | 2009 |
|  | KJ627422 | HMPV/Homo sapiens/PER/FLE7120/2009/A | Peru | 2009 |
|  | KJ627424 | HMPV/Homo sapiens/PER/FLA5834/2009/A | Peru | 2009 |
|  | KJ627426 | HMPV/Homo sapiens/PER/FLA5066/2008/A | Peru | 2008 |
|  | KJ627429 | HMPV/Homo sapiens/PER/CFI1235/2011/A | Peru | 2011 |
|  | KJ627430 | HMPV/Homo sapiens/PER/FLE7557/2009/A | Peru | 2009 |
|  | KJ627434 | HMPV/Homo sapiens/PER/FLA5055/2008/A | Peru | 2008 |
|  | KJ627436 | HMPV/Homo sapiens/PER/FLE7537/2009/A | Peru | 2009 |
|  | KJ627437 | HMPV/Homo sapiens/PER/FPP00726/2011/A | Peru | 2011 |
| Subgroup | Accession # | Strain name | Region | Year |
| B1 | JN184401 | TN982-42 | USA | 1998 |
| (n = 15) | KC562219 | HMPV/USA/C2-175/2005/B | USA | 2005 |
|  | KC562230 | HMPV/AUS/159148534/2004/B | Australia | 2004 |
|  | KC562235 | HMPV/USA/C2-202/2004/B | USA | 2004 |
|  | KC562242 | HMPV/USA/C1-334/2004/B | USA | 2004 |
|  | KF530163 | HMPV/AUS/183399477/2004/B | Australia | 2004 |
|  | KF530164 | HMPV/AUS/172832788/2004/B | Australia | 2004 |
|  | KF530167 | HMPV/AUS/172820414/2004/B | Australia | 2004 |
|  | KF530171 | HMPV/AUS/172431236/2004/B | Australia | 2004 |
|  | KF530173 | HMPV/AUS/183349656/2004/B | Australia | 2004 |
|  | KF530179 | HMPV/AUS/133875417/2003/B | Australia | 2003 |
|  | KJ627383 | HMPV/Homo sapiens/PER/FLA4809/2008/A | Peru | 2008 |
|  | KJ627431 | HMPV/Homo sapiens/PER/FLA6941/2009/A | Peru | 2009 |
|  | KJ627435 | HMPV/Homo sapiens/PER/FLE0425/2009/A | Peru | 2009 |
| Subgroup | Accession # | Strain name | Region | Year |
| B2 | FJ168778 | NL/94/01 | Netherlands | 1994 |
| (n = 16) | JN184402 | TN 99-419 | USA | 1999 |
|  | KC403971 | HMPV/USA/TN-91-316/1991/B | USA | 1991 |
|  | KC403972 | HMPV/USA/TN-91-320/1991/B | USA | 1991 |
|  | KC562227 | HMPV/USA/TN-96-313/1996/B | USA | 1996 |
|  | KC562228 | HMPV/USA/TN-97-235/1997/B | USA | 1997 |
|  | KC562231 | HMPV/AUS/172437891/2004/B | Australia | 2004 |
|  | KC562232 | HMPV/USA/TN-01-28/2001/B | USA | 2001 |
|  | KC562238 | HMPV/USA/TN-96-313/1996/B | USA | 1996 |
|  | KC562239 | HMPV/USA/TN-95-111/1995/B | USA | 1995 |
|  | KC562244 | HMPV/USA/TN-83-1211/1983/B | USA | 1983 |
|  | KF530178 | HMPV/AUS/183219938/2004/B | Australia | 2004 |
|  | KJ627397 | HMPV/Homo sapiens/PER/FPP00098/2010/B | Peru | 2010 |
|  | KJ627400 | HMPV/Homo sapiens/PER/CFI0466/2010/B | Peru | 2010 |
|  | KJ627414 | HMPV/Homo sapiens/PER/CFI0497/2010/B | Peru | 2010 |
|  | KJ627432 | HMPV/Homo sapiens/PER/FLE7218/2009/A | Peru | 2009 |
