# Supplementary material for: Genome-Wide Analysis of Human Metapneumovirus Evolution
Source: PLoS One. 2016 Apr 5;11(4):e0152962. doi: 10.1371/journal.pone.0152962 (PMC4821609; doi:10.1371/journal.pone.0152962)
Supplement: S4 Table — (DOCX) [file pone.0152962.s007.docx]

**S4 Table. Putative recombination strains based on the phylogenetic placements between the pre- and post-breakpoint sequence sets in Fig 4.**

|  |  | Phylogenetic subgroup lineage/sublineage | |
| --- | --- | --- | --- |
| Coding region | Accession No. | Pre-breakpoint region | Post-breakpoint region |
| Complete | KC562241 | A1 | Parental of A2a and A2b |
| F | KC562241 | A1 | A2b |
| SH | JN184399 | A1 | Between A2a and A2b |
|  | KC403976 |  |  |
|  | KC403977 |  |  |
|  | KC403980 |  |  |
|  | KC562226 |  |  |
|  | KC562241 |  |  |
| G | GQ153651 | A2a | A2b |
|  | JN184400 | Parental of A2a and A2b | A2a |
