# Supplementary material for: Genome-Wide Analysis of Human Metapneumovirus Evolution
Source: PLoS One. 2016 Apr 5;11(4):e0152962. doi: 10.1371/journal.pone.0152962 (PMC4821609; doi:10.1371/journal.pone.0152962)
Supplement: S5 Table — (DOCX) [file pone.0152962.s008.docx]

**S5 Table. Recombination breakpoints detected after removing putative recombination strains.**

|  | Potential BP after removing recombination candidates | |
| --- | --- | --- |
| Coding region | Nucleotide | Amino acid |
| F | n.d.^a^ | n.d. |
| SH | n.d. | 153 |
| G | n.d. | 104 |

^a^ n.d., not detected.
