# Supplementary material for: Genome-Wide Analysis of Human Metapneumovirus Evolution
Source: PLoS One. 2016 Apr 5;11(4):e0152962. doi: 10.1371/journal.pone.0152962 (PMC4821609; doi:10.1371/journal.pone.0152962)
Supplement: S6 Table — (DOCX) [file pone.0152962.s009.docx]

**S6 Table. Positive selection codons estimated from the HMPV genomes.**

|  |  |  | Number of positive selection codons | |
| --- | --- | --- | --- | --- |
| Coding region | Lineage | Codon # | SLAC | MEME |
| N | - | 384 | n.d.^a^ | 1 (9)^b^ |
| P | - | 295 | n.d. | 2 (14, 278) |
| M | - | 254 | n.d. | 1 (43) |
| F | Overall | 539 | n.d. | n.d. |
|  | A1 |  | n.d. | n.d. |
|  | A2a |  | n.d. | 1 (179) |
|  | A2b |  | n.d. | n.d. |
|  | B1 |  | n.d. | n.d. |
|  | B2 |  | n.d. | n.d. |
| M2-1 | - | 187 | n.d. | 1 (173) |
| M2-2 | - | 71 | n.d. | n.d. |
| SH | Overall | 177 | n.d. | 3 (9, 64, 104) |
|  | A1 |  | n.d. | 2 (132, 135) |
|  | A2a |  | n.d. | n.d. |
|  | A2b |  | n.d. | 1 (86) |
|  | B1 |  | n.d. | n.d. |
|  | B2 |  | n.d. | 3 (50, 64, 88) |
| G | Overall | 148 | 3 (113, 127, 139) | 5 (113, 121, 127, 139, 143) |
|  | A1 |  | n.d. | 4 (18, 54, 137, 143) |
|  | A2a |  | 1 (146) | 1 (146) |
|  | A2b |  | 1 (102) | 5 (58, 102, 116, 127, 144) |
|  | B1 |  | 1 (109) | 2 (109, 121) |
|  | B2 |  | 1 (113) | 2 (93, 113) |
| L | - | 2050 | n.d. | 11 (8, 32, 68, 422, 677, 1154, 1295, 1336, 1620, 1681, 1888) |

^a^ n.d., not detected.

^b^The amino acid position of selected codons was presented in parenthesis.
