# Supplementary material for: Genome-Wide Analysis of Human Metapneumovirus Evolution
Source: PLoS One. 2016 Apr 5;11(4):e0152962. doi: 10.1371/journal.pone.0152962 (PMC4821609; doi:10.1371/journal.pone.0152962)
Supplement: S7 Table — (DOCX) [file pone.0152962.s010.docx]

**S7 Table. Positive selection codons estimated from the complete genome sequences of HMPVs.**

|  | Codons under positive selection | |
| --- | --- | --- |
| Coding region | SLAC | MEME |
| N | n.d. ^a^ | 9, 51, 210 |
| P | n.d. | 14, 44, 112, |
| M | n.d. | 43 |
| F | n.d. | 166, 518 |
| M2-1 | 173 | 66, 76, 173, 182 |
| M2-2 | 86 | 59 |
| SH | n.d. | 9, 21, 24, 64, 92, 98, 104, 107, 159, 165, 175 |
| G | 102, 113, 127, 139 | 5, 82, 102, 105, 109, 113, 121, 123, 127, 128, 139, 140, 143, 148 |
| L | n.d. | 8, 32, 68, 422, 426, 655, 677, 950, 1154, 1162, 1295, 1336, 1620, 1645 |

^a^ n.d., not detected.
