# Supplementary material for: Genome-Wide Analysis of Human Metapneumovirus Evolution
Source: PLoS One. 2016 Apr 5;11(4):e0152962. doi: 10.1371/journal.pone.0152962 (PMC4821609; doi:10.1371/journal.pone.0152962)
Supplement: S8 Table — (DOCX) [file pone.0152962.s011.docx]

**S8 Table. Estimated time of most recent common ancestors (tMRCA) of the HMPV genomes.**

|  | Mean tMRCA (year) | | | | | |
| --- | --- | --- | --- | --- | --- | --- |
| Gene | Overall | A1 | A2a | A2b | B1 | B2 |
| Complete | 390.92  (154.25-883.62)^a^ | 58.63  (43.39-75.32) | 44.22  (30.28-62.18) | 36.01  (24.71-48.83) | 41.71  (23.15-59.83) | 46.91  (38.30-57.88) |
| N | 255.01  (105.40-414.04) | 31.90  (28.62-37.46) | 33.52  (22.29-46.65) | 23.48  (16.13-33.52) | 29.85  (17.57-43.19) | 34.42  (28.94-40.82) |
| P | 288.46  (91.59-554.66) | 31.84  (28.61-38.43) | 32.21  (21.13-47.29) | 23.67  (14.48-33.86) | 26.74  (15.33-37.83) | 36.50  (28.43-45.94) |
| M | 254.42  (85.85-454.44) | 31.43  (28.61-37.67) | 34.27  (22.26-48.17) | 21.81  (13.43-32.03) | 30.87  (15.90-48.16) | 39.02  (30.65-50.03) |
| F | 289.30  (93.96-584-39) | 70.19  (39.73-104.60) | 33.50  (21.33-48.23) | 25.45  (16.61-36.09) | 34.15  (18.65-54.11) | 36.93  (30.37-46.19) |
| M2 | 224.03  (76.80-381.28) | 54.56  (34.20-80.81) | 30.42  (19.76-44.28) | 16.20  (11.27-21.42) | 25.57  (15.79-42.69) | 37.98  (30.02-48.30) |
| SH | 255.70  (63.10-567.12) | 40.70  (30.98-53.65) | 31.80  (19.77-44.03) | 15.88  (11.30-20.70) | 24.10  (14.69-38.28) | 39.33  (30.18-50.71) |
| G | 352.79  (116.53-687.09) | 75.64  (38.83-121.64) | 31.89  (21.69-41.91) | 29.26  (17.94-41.15) | 31.59  (19.58-45.54) | 36.76  (29.70-46.43) |
| L | 472.45  (136.35-894.67) | 48.80  (35.90-64.32) | 44.52  (28.44-61.99) | 31.45  (18.76-43.68) | 34.87  (19.35-51.21) | 42.74  (33.08-53.29) |

^a^ Lower and upper limits of 95% HPD of the estimated tMRCA are provided in parenthesis.
