# Supplementary material for: Genome-Wide Analysis of Human Metapneumovirus Evolution
Source: PLoS One. 2016 Apr 5;11(4):e0152962. doi: 10.1371/journal.pone.0152962 (PMC4821609; doi:10.1371/journal.pone.0152962)
Supplement: S9 Table — (DOCX) [file pone.0152962.s012.docx]

**S9 Table. Index of relative genetic diversity of the HMPV genomes.**

|  | Index of relative genetic diversity (N*_e_*τ) | | |
| --- | --- | --- | --- |
| Gene | Overall mean | Lowest | Highest |
| Complete | 134.64 | 13.04 (1.78-36.87) ^a^ | 190.64 (81.44-435.28) |
| N | 69.10 | 10.23 (2.30-27.15) | 109.89 (43.60-230.10) |
| P | 71.20 | 9.62 (1.97-32.43) | 109.48 (38.88-283.84) |
| M | 68.06 | 6 (1.33-17.54) | 105.79 (41.77-260.24) |
| F | 88.37 | 4.38 (0.93-11.20) | 113.4 (59.43-244.33) |
| M2 | 53.85 | 6.35 (1.78-18.61) | 66.23 (37.32-151.62) |
| SH | 63.36 | 4.43 (0.85-13.65) | 66.54 (34.68-210.90) |
| G | 94.75 | 5.15 (1.22-14.36) | 124.48 (58.58-247.50) |
| L | 120.26 | 10.94 (2.40-29.27) | 215.79 (88.02-512.15) |

^a^ Lower and upper limits of 95% HPD of the estimated genetic diversity are provided in parenthesis.
